# The Effect of *clpP* Gene Disruption on Cell Morphology, Growth, and the Ability to Synthesize Cellulose of *Komagataeibacter xylinus* E_25_

**DOI:** 10.3390/ijms262412047

**Published:** 2025-12-15

**Authors:** Marzena Jędrzejczak-Krzepkowska, Karolina Ludwicka, Stanislaw Bielecki

**Affiliations:** Institute of Molecular and Industrial Biotechnology, Lodz University of Technology, Stefanowskiego Street 2/22, 90-537 Lodz, Poland

**Keywords:** bacterial cellulose, *Komagataeibacter*, ClpP, biofilm formation, stress tolerance, exopolysaccharides

## Abstract

*Komagataeibacter* species are the best producers of bacterial nanocellulose membranes (BNC)—amazing biomaterial with unique properties and applications in the medical and food industries. The molecular mechanisms of BNC synthesis control remain poorly understood and the need for BNC production and structure improvement is growing. Looking for the genes significant for biosynthesis, we studied the unexplored effect of ClpP proteolytic subunit inactivation on *Komagataeibacter xylinus* E_25_ cell morphology and production of BNC. A mutant with a disrupted *clpP* gene and a complemented strain were obtained. The colonies of the mutant cells, in contrast to the wild-type and complemented ones, were smaller, irregular, and were surrounded by a polymeric noncellulosic envelope. Additionally, the mutant cells were longer and organized in chains, showing different growth and production dynamics of BNC when grown under standard conditions. We also observed worse growth and production of BNC at 5 °C above optimal temperature and in the presence of increased levels of ethanol. E_25_ mutant cells were characterized by lower viability under stress conditions. The 3D microstructure of BNC displayed thicker fibers and denser packing and contained more hard-to-extract exopolysaccharides (HE-EPSs). Based on the outcomes, we conclude that the effect of ClpP on *K. xylinus* decreased resistance to stress and lowered the BNC production level.

## 1. Introduction

Bacterial nanocellulose (BNC) is a natural polymer, insoluble in water, and produced by numerous microorganisms, mainly from the genera *Aerobacter*, *Achromobacter*, *Agrobacterium*, *Alcaligenes*, *Azotobacter*, *Enterobacter*, *Escherichia*, *Gluconoacetobacter* (formerly *Acetobacter*), *Asaia*, *Komagataeibacter* (earlier *Acetobacter*, *Gluconoacetobacter*), *Pseudomonas*, *Rhizobium*, *Salmonella*, and *Sarcina*. Among them, bacteria from the genus *Komagataeibacter*, especially the species *Komagataeibacter xylinus*, *Komagataeibacter sucrofermentans*, and *Komagataeibacter hansenii*, are the model, most efficient producers [1,2]. The natural habitats of these aerobic microorganisms include rotten fruit, vegetables, wine vinegar, fermented beverages, fruit juices, and alcoholic beverages [3,4]. In natural conditions they produce cellulosic biofilm that forms a shield for the cells against the effects of unfavorable environmental factors (e.g., drying out, UV radiation). It facilitates the existence of bacteria in the natural environment and, by allowing them to stay on the surface of a liquid medium (such as juice, wine, etc.), enables bacteria to move and colonize a given ecological niche, ensuring access to and effective transport of nutrients and oxygen [5,6,7]. In laboratory conditions, in liquid stationary culture, cellulose-producing bacteria form a white, leathery membrane (called a pellicle) at the air–liquid interface, composed primarily of cellulose (approximately 90% cellulose by dry weight) and a small amount (from 0.2 to 8.6% of the total BNC dry weight) of hard-to-extract exopolysaccharides (HE-EPS). The composition and amount of HE-EPS in the cellulose membrane depends on the strain and carbon source. HE-EPS are non-cellulosic polymers, insoluble in water but soluble in alkaline media, rich in mannose, and containing glucose and rhamnose. In addition, *Komagataeibacter* species produce polymers such as water-soluble EPS (called acetan, containing D-glucose, D-mannose, L-rhamnose, and glucuronic acid in a ratio of 3–4:1:1:1) [8,9,10]. Ultimately, the total EPS content determines the quality of BNC membranes.

Due to its natural function, BNC is characterized by unique properties (such as a high hydrophilicity, high degree of crystallinity, high purity, and hydration of the material and a lack of cytotoxicity, biocompatibility, and mechanical strength). Owing to these properties and the possibility of biological, chemical, and physical modification, it is used mainly for cosmetic masks and wound dressings. Its suitability for neurotubes, skin substitutes, dental and ear implants, implants of an auricular and cartilage nature, and meshes for hernia surgery was investigated [11,12,13]. Furthermore, it is a useful material for the production of healthy food and for packaging and was proposed for electrical and sensor applications [3,14]. The huge potential of this biomaterial in numerous fields makes industrial-scale production a necessity. Therefore, we look for efficient and cheap methods of BNC biomanufacture and structure control. The manipulation and optimization of cultivation methods has been considered, including principally different carbon sources (such as glycerol, fructose, and agro and food waste products), the presence of stress factors (e.g., ethanol supplementation, oxidative stress, temperature change), and bioreactor design [15,16,17].

Along with a technological approach, research on the molecular mechanisms of cellulose production takes place, giving a new perspective for BNC synthesis and its novel functionalization. It was established that the first main source of altered cellulose production were the mutations in genes directly involved in the biosynthesis process, e.g., genes encoding the BcsB and BcsC subunits of cellulose synthase [18]. These changes include nonsense mutations (single nucleotide insertion or deletion, e.g., in *K. medellinensis* NBRC 3288 and *K. oboediens* MSKU 3, or IS1031 insertion elements (*Ga. xylinus*) [19,20,21,22]). Moreover, it was documented that in addition to key enzymes in cellulose biosynthesis, such as cellulose synthase, endo-b-1,4-glucanase (CMCAx), cellulose-complementing factor (CcpAx), and b-glucosidase (BglAx), BNC yield and crystallinity were influenced by the *alaR* and *ldc* genes, encoding alanine racemase and lysine decarboxylase, respectively [23,24]. Mutants with a disrupted *alaR* or *ldc* gene synthesized less BNC. At the same time the cellulose membranes of mutants were less crystalline and more heterogeneous, containing polymers other than cellulose, composed of galactose and mannose, as compared to the membranes of the parent strain [25]. However, until now the function of the above-mentioned genes was not totally understood; in addition, other fragments of the bacterial genome were examined for their contribution to the BNC biosynthesis process and its regulation.

Beyond bacterial cellulose synthase (BcsABCD) machinery, there are numerous proteins that might play significant roles in BNC biosynthesis, and ClpAPS protease is suggested to be one of them [2]. Clp (EC 3.4.21.92) is an ATP-dependent proteolytic enzyme built of three subunits (A, P, and S), found in many organisms. This complex is known to influence bacterial growth and to play an indispensable role in cellular protein quality control systems, i.e., refolding or degrading damaged proteins in stress conditions, therefore participating in stress tolerance and indirectly in biofilm formation [26]. Laurent et al. analyzed the function of ClpA and ClpS and the interactions between these subunits and BNC production in *Komagataeibacter sucrofermentans*. They showed that the 12-base-pair deletion in the *clpA* gene, coding for the ClpA subunit (responsible for selective binding of proteins to the ClpS subunit), resulted in the formation of cellulose with an exquisite structure and improved mechanical properties [2]. They also noticed that the lack of ClpA-ClpS interactions resulting from the *clpA* gene mutation is insufficient to explain the phenotype they obtained. This indicates the significance of the third subunit, ClpP, in BNC formation, but this still remains unexplored. This third, proteolytic part of Clp is known as a serine protease (containing a serine–histidine–aspartate catalytic triad) involved in various stress responses [27]. ClpP, the catalytic core of the Clp proteolytic complex, takes part in cellular protein quality control. To survive in stress conditions, cells synthesize proteins, including chaperones and proteases. These stress proteins are also involved in various cellular regulations during growth also in biofilm formation. Along with ATPases, ClpP identifies, unfolds, and degrades damaged or misfolded proteins, preventing them from accumulating and becoming toxic to the cell [28,29]. The role of ClpP described in the literature, however, concerns mainly pathogenic bacteria such as *Enterococcus faecalis*, *Actinobacillus pleuropneumoniae*, *Haemophilus parasuis*, *Porphyromonas gingivalis*, *Staphylococcus epidermidis*, *Staphylococcus aureus*, or *Pseudomonas aeruginosa* [26,29,30,31,32,33,34,35]. In cellulose-producing *Komagataeibacter* strains, which are constantly exposed to acidic, oxidative, and ethanol stress during surface cultivation, ClpP is expected to participate in protective mechanisms by removing misfolded or damaged proteins and by modulating the balance between stress survival and energy-intensive BNC biosynthesis, thereby supporting long-term pellicle formation and overall cell fitness. However, most functional studies on ClpP reported so far have focused on pathogenic bacteria, where this protease promotes stress survival, regulates virulence factor production and biofilm formation, and contributes to tolerance to multiple antibiotics [26,29,30,31,32,33,34,35]. These models, while highly informative for the general biology of ClpP, do not address its specific role in cellulose-producing acetic acid bacteria.

In the provided article we investigate the effect of *clpP* gene disruption on cell morphology and the BNC biosynthesis process in a nonpathogenic, cellulose-producing microorganism, specifically in *Komagataeibacter xylinus* E_25_. To our knowledge, this is the first study that directly examines the consequences of a targeted *clpP* disruption for BNC biosynthesis in a *Komagataeibacter* strain. The main goal was to understand the mechanisms of cellulose biosynthesis and its regulation, as well as the mechanisms of cell protection against chosen stress factors relating to the presence of ClpP protease. By analyzing bacterial growth and biofilm formation, as well as selected features of the produced cellulose, we infer the role of ClpP in the microbial metabolism of a cellulose-producing organism and provide initial insight into its impact on cellulose quality.

## 2. Results

### 2.1. Disruption and Complementation of clpP Gene in K. xylinus E_25_

To investigate the role of ClpP in environmental stress tolerance and biofilm formation in *K. xylinus*, gene-disrupted and complementation mutants were obtained. The method of homologous recombination was applied, as described in Section 4.4. Genomic DNA was isolated from the selected recombinant, and the disruption of the *clpP* gene was confirmed by PCR and electrophoretic analysis (Figure S1). Finally, the sequencing of PCR products confirmed the presence of the mutant strain with a disrupted *clpP* gene (E_25_ clpP:amp mutant). The presence of the E_25_C complementation strain was confirmed by the same procedure and plasmid DNA isolation and sequencing, as well as subsequent protein analysis via Western blot (Figure S2). A protein band similar to the molecular weight of ClpP (25.56 kDa) was obtained for E_25_C but not for the wild-type strain or the E_25_ clpP:amp mutant. These results indicate the successful generation of a complementation strain.

### 2.2. Morphology of Colonies and Cells of E_25_ Strain and Its Mutants

**Figure 1:**
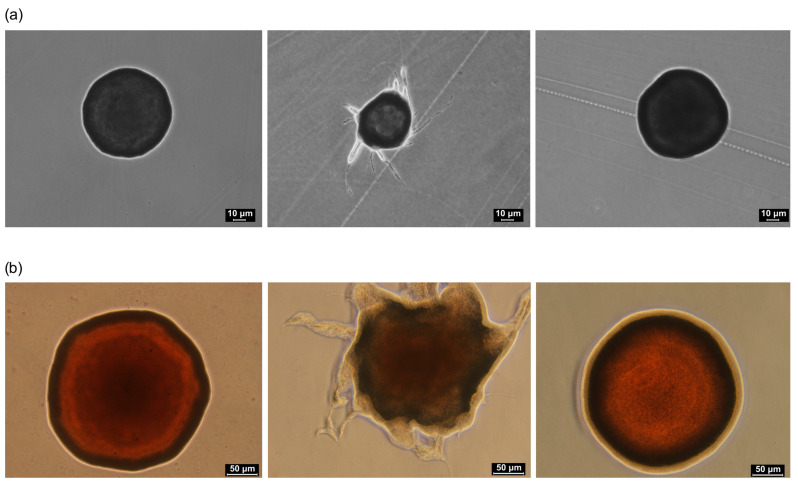
Microscopic images of *K. xylinus* E_25_ colonies (control sample), E_25_ clpP:amp, and E_25_C (**a**) on HS agar medium (magnification 100×) and (**b**) on HS agar medium supplemented with Congo red (magnification 200×).

**Figure 2:**
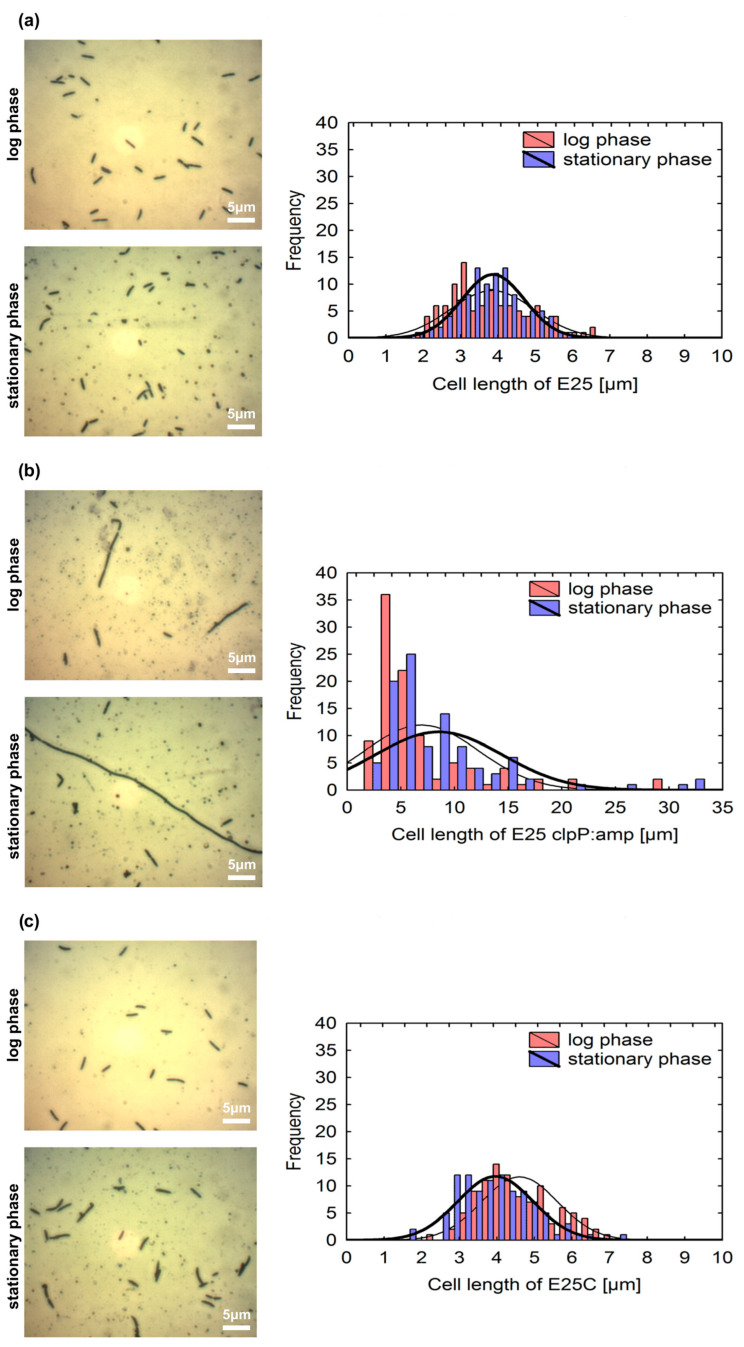
Cell morphology of strains (**a**) E_25_, (**b**) E_25_ clpP:amp, and (**c**) E_25_C; left column demonstrates microscopic images of cells stained with crystal violet, visualized by light microscopy in log and stationary phases after 2 and 3 days of culture; right column presents the graphs describing the distribution frequency of cell lengths (100 cell measurements were made for each sample).

**Figure 3:**
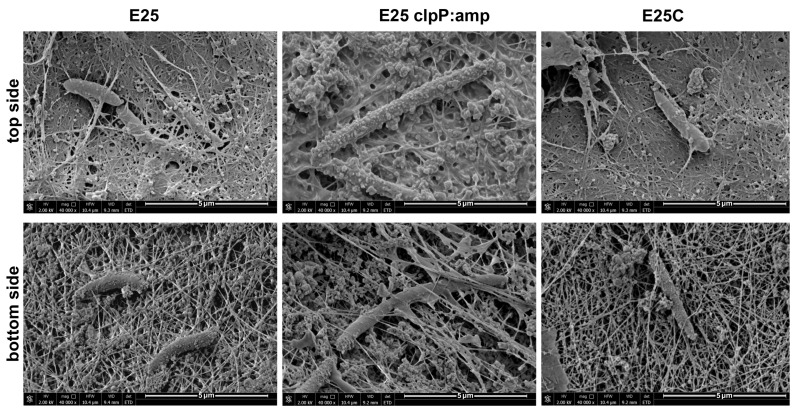
SEM images of E_25_, E_25_ clpP:amp, and E_25_C biofilms from the top and bottom side of the membranes after 3 days of culture.

In the next step we investigated whether ClpP affected colony or cell morphology. All the strains, E_25_, E_25_ clpP:amp, and E_25_C, were observed using optical and electron microscopy techniques (Figure 1a,b). It was found that on solid HS medium, the colonies of the wild-type strain and E_25_C were similar in size and shape (spherical and smooth), in contrast to colonies of the E_25_ clpP:amp mutant, which were smaller and had a filamentous margin and an irregular polymeric envelope. The polymer surrounding E_25_ clpP:amp colonies was not cellulose, as it was not stained red on HS medium supplemented with Congo red (Figure 1b). It is known from the literature that Congo red has a strong affinity for cellulose, and the degree of staining with this ionic azo dye can be used to estimate the strain’s ability to produce cellulose [1]. Further, we determined that a single colony of E_25_ clpP:amp mutant was formed by a smaller number of cells (2.46 × 10^6^ ± 0.9 × 10^6^ CFU/mL) as compared to the wild strain (5.9 × 10^7^ ± 1.67 × 10^7^ CFU/mL). From the optical microscope observations, it can be seen that the cells of the E_25_ clpP:amp mutant were longer (average length of 9 μm) and often organized in chains (Figure 2). By applying SEM, the cells were also observed when caught within a biofilm. The obtained images visualize the impaired cell division in the case of the E_25_ clpP:amp mutant. Moreover, E_25_ clpP:amp cells show a more irregular surface; they are rough, which is especially visible in the SEM analyses of the upper part of the biofilm (Figure 3).

### 2.3. The Profile of Growth and BNC Production by Native and Mutant Strains

**Figure 4:**
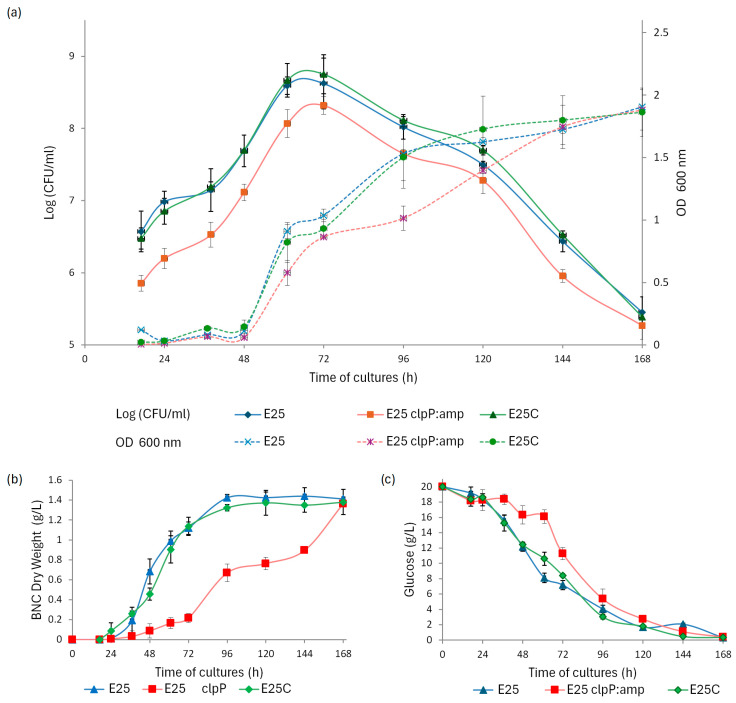
(**a**) The dynamics of bacterial growth; (**b**) the amount of cellulose synthesized in time as related to (**c**) glucose concentration culture medium for the native, mutant, and complementation strain.

In the first stage the research was focused on the effect of clpP gene disruption on bacterial growth, BNC biosynthesis, and glucose consumption under optimal culture conditions. The obtained results are presented in Figure 4. Bacterial growth was determined using two methods: the spread plating method and the measurement of optical density (OD_600_) of culture medium after the degradation of BNC by cellulase. The E_25_ clpP:amp strain was observed to grow more slowly than the wild-type one, with the number of viable cells approximately one order of magnitude lower than the number of cells in the parent strain and E_25_C. The E_25_ and E_25_C strains reached stationary phase after 61 h of culture, while the E_25_ clpP:amp mutant reached stationary phase 12 h later (Figure 4a). OD_600_ for all three strains was comparable up to 72 h; after that time, OD started to grow faster for E_25_ and the E_25_C mutant until after 144 h it became again comparable with E_25_ clpP:amp. The OD increase was most probably disrupted by turbidity appearing as a result of constant cellulose production by all bacteria. At the same time we observed changes in the dynamics of glucose consumption and cellulose biosynthesis by all three strains (Figure 4b,c). The E_25_ clpP:amp mutant consumed glucose more slowly until 96 h; at 48 h and 72 h, glucose concentrations in the culture medium were higher by approximately 26% and 36%, respectively, compared to the control strains. Glucose consumption by the strains correlated with the rate of BNC biosynthesis. The E_25_ clpP:amp mutant produced cellulose significantly slower compared to the parent strain and the complementation mutant. Furthermore, as visible in Figure 4b, the wild-type strain and E_25_C began to intensively produce BNC membrane from the 36th hour in the early log growth phase and had already reached the highest BNC dry weight value on the 4th day, while E_25_ clpP:amp produced approximately half of that during this time. Interestingly, after the 4th day of incubation, E_25_ and E_25_C produced BNC at similar levels, whereas E_25_ clpP:amp achieved a comparable level of BNC dry matter production to E_25_ and the complementation strain on the 7th day. These results indicate that ClpP is required for BNC biosynthesis.

### 2.4. Stress Tolerance

**Figure 5:**
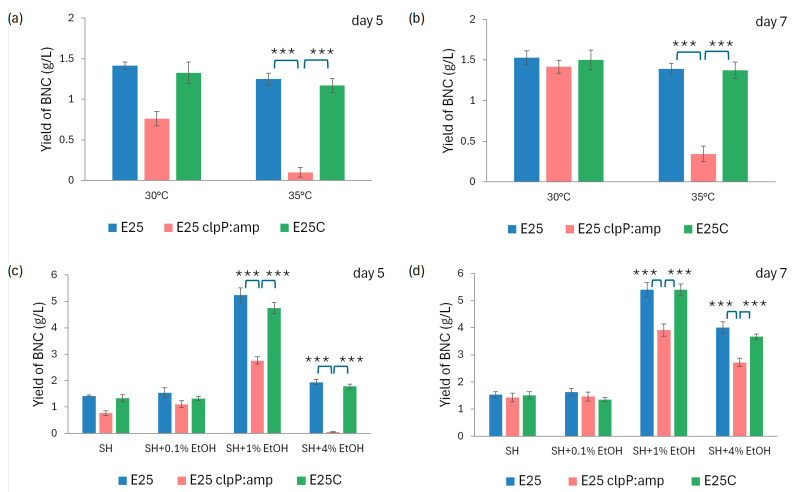
The influence of stress conditions on bacterial growth and BNC synthesis yield: (**a**) effect of temperature after 5 days and (**b**) 7 days of culture. (**c**) Effect of ethanol addition after 5 and (**d**) 7 days of culture. Statistical significance: *** *p* < 0.001. Error bars represent standard deviations.

In order to determine the role of ClpP protease in the mechanisms of tolerance to environmental stress and in biofilm formation by *K. xylinus*, in the next step we studied the effect of *clpP* gene disruption on BNC biosynthesis under the influence of stress factors: temperature (bacteria culture at a temperature 5 °C higher than optimal) and hydrogen peroxide and ethanol presence (medium supplementation). The results of these analyses are collected in Figure 5. It was observed that E_25_ clpP:amp synthesized BNC at a lower level at a temperature 5 °C higher than the optimal one compared to the control native strain (Figure 5a,b). The biosynthetic efficiency of E_25_ clpP:amp on the 5th and 7th day of culture was only 14.3% and 23% of the efficiency of E_25_ or E_25_C, respectively. The obtained results indicate an important role of ClpP in BNC production in response to temperature shock. Among the tested ethanol concentrations, the addition of 1% EtOH to the medium was the most optimal for the tested strains in terms of BNC biosynthesis (Figure 5c,d). On the 7th day, it caused a 3.5-fold increase in BNC biosynthesis for E_25_ and E_25_C and a 2.5-fold increase for E_25_ clpP:amp as compared to the absence of ethanol supplementation in HS medium. At the same time, after 7 days of culture with 1% addition of EtOH to HS medium, the E_25_ clpP:amp mutant produced 27% less BNC than E_25_ and E_25_C. The obtained results confirm that ClpP is needed for the growth of BNC and its formation process in the presence of ethanol.

**Figure 6:**
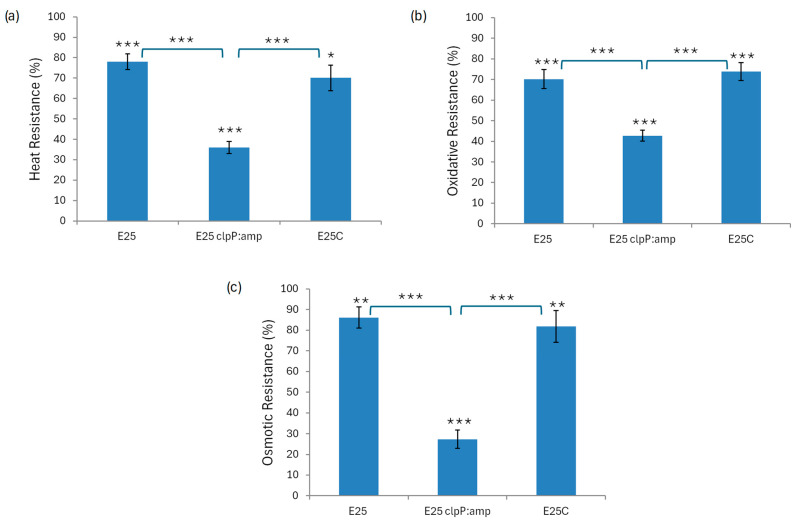
Analysis of stress tolerance of the strains to elevated temperature (**a**), oxidation (**b**), and osmotic pressure (**c**). Statistical significance: * *p* < 0.05, ** *p* < 0.01, *** *p* < 0.001. Error bars represent standard deviations. The stars above the bars represent the comparison of stress-treated samples with control samples (not exposed to the stressor).

Finally, E_25_, E_25_ clpP:amp, and E_25_C cells in log-phase growth were subjected to various stress factors, such as heat shock, oxidative stress, and osmotic stress. When the cells were incubated at 40 °C for 10 min, the heat-shock resistance of the E_25_clpP:amp mutant was 2-fold lower than for the wild-type strain and E_25_C (Figure 6a). These results demonstrate that ClpP is involved in the response of *K. xylinus* to heat changes. Similar results were obtained in oxidative stress assays, where E_25_ clpP:amp mutant cells incubated in the presence of 0.5 mM H_2_O_2_ for 30 min showed 1.6-fold lower survival rates as compared to the survival of wild-type and complementation cells under the same conditions (Figure 6b). Analysis of cell tolerance to 0.3 M KCl for 1 h incubation showed that the resistance of the wild-type strain to osmotic stress was similar to that of the complementation mutant E_25_C and 3-fold higher as related to the survival rate of E_25_ clpP:amp (Figure 6c). These results indicate that ClpP also plays an important role in osmotic stress tolerance.

### 2.5. Morphology of Cellulose Membranes

**Figure 7:**
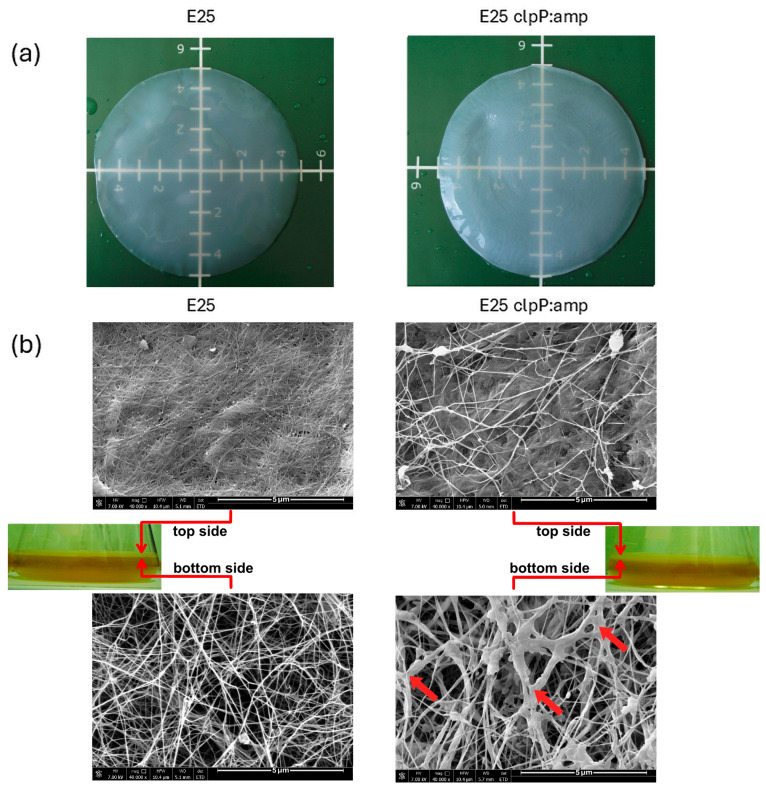
Visualization of BNC pellicles produced from glucose by *K. xylinus* E_25_ and E_25_ clpP:amp mutant after 7 days of culture: (**a**) photographs in macroscale; (**b**) SEM images showing the morphology and arrangement of fibers from the top and bottom side of the membranes; The red arrows indicate thickened fibers, which represent a difference compared to the fibers synthesized by the *K. xylinus* E_25_ strain.

After identifying the role of ClpP in bacterial growth and cellulose biosynthesis, the morphology of BNC membranes was analyzed. The photographs presented in Figure 7a demonstrate the transparency increase in the pellicles after clpP disruption. Also, the microarchitecture of BNC membranes examined by SEM was changed (Figure 7b). SEM images showed that BNC synthesized by E_25_ clpP:amp was composed of fibers with more variable thickness as compared to E_25_ membranes. Interestingly, it was found that BNC membranes produced by a mutant strain consisted of a network of fibers containing, in addition to cellulose chains, non-cellulosic polysaccharides (exopolysaccharides, EPSs), among which a hard-to-extract fraction (HE-EPS) constituted almost 3% of the dry weight of BNC (Table 1). Looking at the SEM pictures (Figure 7b), one may notice the entangled cellulose fibers looking as if they were covered with a sticky substance. Due to its presence the membrane microstructure is denser than what can be seen in a macroscale (Figure 7a). Exopolysaccharides coproduced with pure cellulose attach to the fibers, changing the membrane’s appearance, structure, and, undoubtedly, properties. However, detailed research in this area will be the subject of the upcoming article.

## 3. Discussion

Bacterial nanocellulose has been one of the most prospective biomaterials in medicine and in many sectors of industry. Due to its incredible potential, the improvement of biosynthesis efficiency has been one of the greatest challenges for the researchers working with this unique biopolymer. At the same time, working on the BNC structure and its modification and improvement gives the opportunity to expand the scope of applications and adjust its properties to specific goals. The molecular approach exploiting specific genes participating in cellulose biosynthesis could possibly allow enhanced biosynthesis and specific, directed manipulations of BNC structure. In the presented study, we focused on the *clpP* gene which, in other bacterial species, was already recognized to participate in biofilm formation and adaptation to various environmental conditions. During our study we generated a mutant strain of *K. xylinus* E_25_ with a disrupted *clpP* gene and a complementation strain to confirm the obtained phenotypic changes in the investigated gene and verify if the observed changes were actually caused by this mutation. In the first stage we analyzed the effect of this mutation on the morphology of bacterial cells and the colonies they form in standard conditions. We observed that E_25_ clpP:amp cells were rougher (had an irregular surface area (Figure 3)) and had an average cell length 2.5 times greater than E_25_ or E_25_C cells (Figure 2). Our results indicate that ClpP directly or indirectly participates in cell division and plays an important role in maintaining the cellular morphology of *K. xylinus*. The lower growth rate of E_25_ clpP:amp during the culture, expressed as CFU/mL, may result from impaired cell division. As described in the literature, ClpP is essential for bacterial growth and influences cell division, but the changes it causes depend on the bacteria analyzed. Deletion of the *clpP* gene in *Haemophilus parasuis* resulted in shorter cell formation [31]. In *Actinobacillus pleuropneumoniae*, mutant cells lacking ClpP were also shorter, but they were rougher, characterized by an irregular surface and a 1.8-fold increase in volume [30]. For the *clpP* deletion mutant of *Porphyromonas gingivalis*, the cells had a smoother surface and more regular shape [32]. In contrast, in *Caulobacter crescentus*, the disruption of the *clpP* gene resulted in longer cell formation. To date, ClpP has been shown to be involved in cell cycle control, as the substrate for its proteolytic activity is the regulatory protein CtrA (a transcription factor and element of two-component systems). The presence of CtrA arrests cells in the G1 phase of the cell cycle and prevents them from entering mitosis [36]. Knudsen et al. reported the effect of ClpP on another post-transcriptional regulator CsrA that controls a large variety of physiological processes such as central carbon metabolism, motility, and biofilm formation [37]. Nevertheless, it is hard to explain the interactions of ClpP as related to differing effects between various strains since the information in this field is barely described in the literature. Bacterial biofilm formation is a complex process that requires the participation of numerous genes responsible for adhesion, metabolism, quorum sensing, and stress response. It may be presumed that ClpP may simultaneously disrupt multiple related regulatory networks by affecting the transcriptome or protein stability [29].

The outcomes we describe are the first to demonstrate the role of ClpP in the formation of cellulose membrane. The disruption of the *clpP* gene was found to slow down cellulose biosynthesis in all analyzed culture conditions (see Figure 4 and Figure 5). ClpP is known to have an impact on the dynamics of biofilm formation in bacteria, exhibiting either inhibitory or stimulating effects. A reduction in biofilm formation was observed in ClpP-deficient mutants in *Pseudomonas fluorescens*, *Streptococcus* mutants, *Actinobacillus pleuropneumoniae*, *Porphyromonas gingivalis*, and *Staphylococcus epidermidis* [30,32,33,38,39]. However, an increase in biofilm formation was observed in ClpP-deficient mutants of *Haemophilus parasuis* and *Staphylococcus aureus* [31,40]. Based on these findings, it was hypothesized that ClpP protease may be responsible for the initial stage of biofilm formation, i.e., the association of bacteria with the substrate [33,38]. The proteolytic subunit of ClpP relies on proteins as substrates. For instance, after the disruption of the *clpP* gene in *Staphylococcus aureus*, deposits of denatured, self-associating proteins were found in mutant cells [41,42]. This effect most likely arises because the damaged proteolytic unit prevents the other subunits cooperating with ClpP from performing their functions related to selective protein degradation. We also know that the proteins regulating cell-signaling processes, e.g., phosphodiesterase A (PDEA), can be the substrates for ClpP. For instance, the ClpXP complex has been shown to degrade PDEA in *Caulobacter crescentus* [38,39]. In turn, PDEA degrades c-di-GMP, which controls many bacterial life processes and, most importantly, is an allosteric activator of the cellulose synthase complex responsible for BNC biosynthesis in *Komagataeibacter* [43,44]. There is no information in the literature on the effect of ClpP on cellulose biosynthesis in *Komagataeibacter*, but the results obtained for *C. crescentus* suggest that the E_25_ clpP:amp mutant may show limited PDEA degradation and, consequently, faster c-di-GMP degradation and lower BNC biosynthesis efficiency. It is unknown whether limited PDEA degradation occurs in E_25_ clpP:amp cells and, if so, whether it has a significant impact on biosynthesis, but our study on disruption mutants showed that the cells lacking a functional *clpP* gene visibly synthesized less cellulose.

The second part of our research concerned the analysis of growth and cellulose biosynthesis of the mutant strain when exposed to various stress factors (Figure 5 and Figure 6). Hydrogen peroxide (which causes oxidative stress), KCl (osmotic stress), and temperature (thermal shock) are frequently used in the literature to study the effects of stress factors on bacterial growth and viability [26,29,30,45]. González-García et al. observed an inhibition of cellulose growth in the presence of NaCl (5 g/L and 20 g/L) [46]. At the same time the level of biomass production was significantly lower and even residual for the higher concentration. The authors speculated that some bacteria in such conditions are not able to consume the nutrients in an efficient way. The cells neither reproduce nor produce cellulose to maintain the energy for surviving. Based on this research we applied lower concentrations of salt solution so as not to stop the growth completely. During the study we observed that E_25_ clpP:amp cells were more sensitive to all stress factors analyzed and their viability was reduced as related to the wild-type and complementation strains (Figure 6). We noticed worse BNC production by E_25_ clpP:amp when cultured at a temperature 5 °C above the optimal one (Figure 5a), as well as in a medium supplemented with ethanol (Figure 5b). For *K. xylinus* E_25_ when ethanol is present in the culture medium in a concentration of 1% *v*/*v*, glucose metabolism is directed towards cellulose production due to the induction of genes related to UDP–glucose formation and the repression of genes involved in glycolysis and acetan biosynthesis [47]. However, at concentrations above this value, it starts to inhibit the synthesis of cellulose (Figure 5b). These outcomes indicate that ClpP is needed to maintain the tolerance and adaptation of *K. xylinus* E_25_ to the chosen stress conditions. *ClpP* gene expression has already been associated with environmental stress, since ATPases cooperating with ClpP also act as chaperones and are essential for bacterial survival under both elevated and reduced temperature conditions. Strong proteolytic activity of ClpP was detected in *Staphylococcus aureus* under heat and oxidative shock conditions [41].

The last part of our study was related to the examination of post-culture medium and BNC membrane composition. We observed that E_25_ clpP:amp synthesized 18.5% more acetan (free EPS, Table 1) on the 7th day of culture compared to the wild-type strain. Acetan is a known BNC biosynthesis stimulator. Ishida et al. showed that a mutant with a disrupted *aceA* gene did not synthesize acetate, which resulted in a decrease in cellulose biosynthesis efficiency [48]. It can be assumed that the growing content of acetan promotes the synthesis of cellulose and, in fact, such a growth in production for a mutant strain was observed after 6 days of cultivation (see Figure 3). At the same time, acetate increases viscosity, which makes cell-binding to cellulose fibers more difficult. Therefore, it affects the formation of cellulose ribbons and thus alters the ribbon formation and bundling process [8,49]. Therefore, a greater amount of acetone may influence the crystallinity of BNC and its structure. In our research we observed that the 3D structure of BNC from the mutant strain differs from that synthesized by the wild-type E_25_ (Figure 7). The fiber arrangement in BNC may be influenced by the lower amount of acetate in the post-culture fluid and may also be the result of the appearance of longer cells. During cell division, cellulose fibrils are continuously synthesized, and during cytokinesis, they are distributed among daughter cells, resulting in the formation of a node with three branches. Cellulose membrane synthesized by E_25_ clpP:amp mutant had thicker fibers and a higher density as compared to E_25_ and E_25_C (see Figure 7), possibly as a result of cell division and a more heterogeneous nature of fibers. It was demonstrated that the mutant-synthesized cellulose membranes had an almost 1.4-fold higher efficiency of HE-EPS. Since acetan and all HE-EPSs become the components of BNC membrane, their presence and composition are significant factors, especially from the point of view of physical and physicochemical cellulose properties [9,10]. It may be expected that the denser membrane, containing more EPSs, will demonstrate definitively different mechanical properties, water-holding capacity, or binding capacity, which, in turn, may extend or change the range of applications of such a biopolymer.

To summarize, our findings provide the first direct genetic evidence for a role of ClpP in a cellulose-producing bacterial strain. They give an insight into understanding the protective mechanisms of *Komagataeibacter* cells induced by exposure to stress factors, but we still need further research to confirm the relationships of these processes with BNC structure and properties, as well as to define more precisely the mechanisms of ClpP regulation and its role in cellulose biosynthesis. These data will serve as a basis for further, detailed experimental verification of the significance of the observed differences (e.g., in structure and EPS) for better understanding of the molecular biology of BNC producers.

## 4. Materials and Methods

### 4.1. Chemicals

All chemicals used in the experiment were of analytical grade. They were purchased from Sigma Aldrich Chemie GmbH (Buchs, Switzerland), Promega Corporation (Madison, WI, USA), Chempur (Piekary Śląskie, Poland), and POCh (Gliwice, Poland) unless stated otherwise.

### 4.2. Bacterial Strains, Plasmids, and Culture Conditions

Bacteria, plasmids, and starters applied in this work are listed in Table S1. Bacteria to be used were kept at −70 °C. The *E. coli* DH5α strain as a cloning host organism was cultivated at 37 °C in LB medium supplemented relating to the recombinant plasmid-carrying strain (ampicillin 50 μg/mL or chloramphenicol 30 μg/mL) [50].

*K. xylinus* E_25_, its mutant with the disrupted *clpP* gene (clpP:amp), and the complementation strain (E_25_C) were standardly cultivated in stationary conditions in Hestrin and Schramm (HS) medium of pH 5.7 at 30 °C, as described in earlier published materials [8]. At the beginning all three strains were activated from stock by culturing in HS medium supplemented with 2% agar in Petri dishes at 30 °C for 5 days. A single colony was then inoculated into 5 mL of liquid HS medium and incubated at 30 °C for 3 days (preculture). Standard culture was performed in 500 mL Erlenmeyer flasks filled with 100 mL of liquid HS medium. The medium was inoculated with the preculture of each strain separately in a volume of 5% *v*/*v* and incubated for 7 days at 30 °C in stationary conditions. HS medium was supplemented with antibiotics depending on the mutant strain (Amp 200 μg/mL or Cm 170 μg/mL).

### 4.3. BNC Purification

When needed, BNC membranes were purified of residual media and bacterial cells using standard methods, by rinsing the membranes in water until they became white, then immersing them in 1% NaOH *w*/*v* solution for 24 h. Next, the membranes were washed with 1% *v*/*v* acetic acid for 2 h and finally immersed in water until the pH was neutralized.

### 4.4. Construction of Mutants with Disrupted clpP Gene and Its Complementary Strain

Primers used to inactivate the *clpP* gene in chromosomal DNA and for complementation are listed in Table S1. The *clpP* gene was amplified using clpPF and clpPR primers, Pfu polymerase (Promega Corporation, Madison, WI, USA), and *K. xylinus* E_25_ genomic DNA. The PCR product was cloned into the pET14b vector at the BspHI site, and the resulting plasmid was named pET14bclpP. This plasmid was then cleaved by the BssHII restriction enzyme, and the ampicillin-resistant cassette was inserted into this site by blunt-end ligation. The ampicillin-resistance cassette was amplified using Pfu DNA polymerase (Promega Corporation, Madison, WI, USA), primers AmpF/BssHII and AmpR/BssHII, and the pUC19 vector as a template. The obtained clpP knockout plasmid (pET14bclpP-ampR) was transformed into *K. xylinus* E_25_ by electroporation under conditions described previously [46]. The resulting mutant with an inactivated *clpP* gene in chromosomal DNA (E_25_ clpP:amp) was selected based on its ampicillin resistance. Furthermore, the disruption of the *clpP* gene was confirmed by PCR. The upstream (425 bp) and downstream (383 bp) fragments of *clpP* in the genome were amplified using flankF and flankR primers (see Table S1), Pfu DNA polymerase, and genomic DNA from either the E_25_ clpP:amp mutant or wild-type strain. The obtained PCR product was analyzed on an agarose gel and by sequencing at Genomed S.A. (Warsaw, Poland).

The complementation plasmid was constructed by PCR amplification of the 650 bp clpP gene. The reaction was carried out using Pfu polymerase (Promega Corporation, Madison, WI, USA), primers clpPHF and clpPHR, and *K. xylinus* E_25_ genomic DNA. The pBBR122 vector was digested with XhoI and HindIII restriction enzymes, and the *clpP* gene was inserted into these sites, yielding the pBBR122-clpP vector. The resulting vector was electroporated into the E_25_ clpP:amp mutant under conditions described previously [51]. The complementation mutant E_25_C was selected by resistance to ampicillin and chloramphenicol and by plasmid DNA isolation and sequencing at Genomed S.A. Furthermore, overexpression of ClpP, containing a C-terminal histidine tag, was identified by Western blot. For this purpose, 1 mL samples from cultures of the wild-type E_25_ strain and the E_25_C mutant were collected in HS medium supplemented with 1% cellulase (OD_600_ ~ 0.7) and centrifuged at 5000× *g* for 15 min at 4 °C. Intracellular proteins were extracted using BugBuster Master Mix (Millipore Sigma, Darmstadt, Germany). Proteins contained in cell-free extracts were separated by SDS-PAGE and electro-transferred to a nitrocellulose membrane (pore size of 0.2 μm). The recombinant ClpP was detected using a His–Tag AP Western Reagent and His–Tag Monoclonal Antibody (Merck KGaA, Darmstadt, Germany). Molecular weight of the recombinant ClpP was determined using a Perfect Protein Western Marker (Millipore Sigma, Darmstadt, Germany).

### 4.5. Microscopic Observations of Bacterial Colony Morphology

Bacteria were cultured in Petri dishes on HS medium with 2% agar or HS medium with 2% agar supplemented with Congo red (20 µg/mL) to visualize the cellulose surrounding the cells. The cultures were cultivated at 30 °C for 5 days. Microscopic images were taken using an ECLIPSE TS-100/100F inverted microscope (Nikon Corporation, Tokyo, Japan).

### 4.6. Determination of the Number of Cells Forming a Single Bacterial Colony

A single bacterial colony was collected from a 5-day culture on HS medium with 2% agar, suspended in 100 µL of physiological saline containing 1% cellulase (cellulase from *Trichoderma reesei*, Sigma Aldrich Chemie GmbH, Buchs, Switzerland) and incubated at 30 °C for 1 h at 90 rpm. The resulting suspension was diluted in physiological saline and plated on HS medium with 2% agar. After incubation (at 30 °C for 5 days), the number of colonies grown on the plates was counted, and taking the dilution into account, the number of bacterial cells in 1 mL of physiological saline was calculated. The final result was expressed in CFU/mL.

### 4.7. Microscopic Observations of Bacterial Cells

Cells of the wild-type E_25_ strain, E_25_ clpP:amp, and E_25_C were harvested after 2 and 3 days of culture in HS medium supplemented with cellulase at a final concentration of 1% *v*/*v* (cellulase from *Trichoderma reesei*, Sigma Aldrich Chemie GmbH, Buchs, Switzerland)). Cellulase was added 2 h before the end of incubation. Microscopic observations of cell morphology were performed using a light microscope OPTA-TECH MB 200 (OPTA-TECH, Warsaw, Poland). Microscopic slides were fixed, stained with crystal violet, and observed under 1000× magnification with immersion oil. Cell lengths of the wild-type strain and its mutants were also measured using OptaView-IS software (OptaView-IS, version 3.6.6). For all samples, a minimum of three biological replicates and three experimental replicates were performed. The lengths were measured for least 10 cells randomly chosen from each picture.

### 4.8. Bacterial Growth Assay

Since the bacteria-synthesizing cellulose live not only in the culture medium but also inside the membrane, it is important to release the cells from the surrounding biofilm. Therefore, twenty four hours before the end of cultivation (see point 4.2) at each time point, cellulase at its final concentration of 1% (*v*/*v*) was added to the bacterial culture to enzymatically degrade cellulose the membrane. The resulting bacterial suspension was diluted and used to visualize the microbial growth and calculate bacterial titer

- Spectrophotometrically—bacterial growth was measured using a 6300 VIS/6305 UV–VIS spectrophotometer (Jenway, Stone, Staffordshire, UK) at a wavelength of 600 nm against the control sample (HS medium).
- By the Dip-plating method—100 µL of the diluted 10^1^ to 10^7^ suspension was spread onto Petri dishes containing HS medium with 2% agar. The plates were then incubated for 5 days at 30 °C. After incubation, the number of colonies grown on the plates was counted, and the number of bacterial cells per ml of culture was calculated based on the dilution. The final result was presented in CFU/mL.

### 4.9. Glucose Concentration Determination

Glucose concentration in the post-culture solution was determined using the enzymatic glucose oxidase—peroxidase (GOD-POD) test (BioMaxima, Lublin, Poland), according to the producer’s instructions. Briefly, 1 mL of the GOD-POD test was added to 10 µL of post-culture liquid, appropriately diluted to achieve an absorbance of 0.1 to 0.9. The mixtures were incubated for 15 min at room temperature. After incubation, the absorbance at 500 nm was measured against a blank sample containing distilled water instead of culture liquid. Experiments were performed in triplicate to determine the amount of unused glucose in the medium.

### 4.10. Effect of Temperature and Ethanol on Cell Growth and Cellulose Biosynthesis

Bacteria were cultured at 35 °C or with the addition of ethanol (final concentration in the medium: 0.1, 1, or 4% (*v*/*v*)) under standard conditions. Each strain was cultured in parallel to determine the dry weight of purified cellulose membranes and to verify microbial growth. Experiments were performed in triplicate.

### 4.11. Determination of Bacterial Cell Resistance to Stress

Stress resistance of *K. xylinus* cells was determined by the exposition of cultures to chosen factors. First, 20 mL of bacteria from liquid HS media supplemented with 1% cellulase without shaking were collected (the cells were in logarithmic-growth phase). The culture portions were centrifuged at 5000× *g* and 4 °C for 30 min. Then they were washed with 0.9% NaCl and finally suspended in 10 mL of 0.9% NaCl. To determine the response of bacteria to heat shock, 0.5 mL of cells suspended in physiological saline was taken and added to 0.5 mL of SH and then incubated for 10 min and at 40 °C. To determine the response to oxidative stress, 0.5 mL of cells suspended in saline was added to 0.5 mL of HS medium containing 0.5 mM H_2_O_2_ solution. It was then incubated for 30 min at 30 °C. To determine the response to osmotic shock, 0.5 mL of cells suspended in saline was added to 0.5 mL of HS medium containing 0.6 M KCl and incubated for 1 h at 30 °C. For each strain, a control sample (0.5 mL of cells suspended in saline mixed with 0.5 mL of HS medium) was processed the same way. After incubation the samples were diluted and spread on the plates containing HS medium and 2% agar to determine CFU. Based on the results, stress resistance to chosen factors was calculated (CFU_stressed sample_ m^−1^/CFU_control sample_ ml^−1^) × 100. Experiments were performed in triplicate.

### 4.12. BNC Yield Determination

Purified cellulose membranes were dried at 60 °C and weighed to obtain a dry weight. The final dry weight of BNC was calculated as the dry weight of cellulose obtained from 1 L of culture. The BNC production yield was measured as the dry weight of cellulose per unit volume of medium (g/L).

### 4.13. Scanning Electron Microscopy (SEM) of BNC

The biofilm obtained after 3 days of bacterial cultivation in HS medium was washed and fixed according to the previously described method [52]. To examine the BNC morphology, the water in the purified membranes was replaced with 30 to 100% (*v*/*v*) 2-propanol solutions. The samples were then dried using an Alpha 1-4 LSC freeze-dryer (Martin Christ Gefriertrocknungsanlagen GmbH, Osterode am Harz, Germany). The dried membranes were coated with gold using a sputter coater (Leica AM ACE600 (Leica Microsystems GmbH, Wetzlar, Germany)). BNC surface analysis was performed using a Quanta FEG 250 FEI scanning electron microscope from FEI Company, Hillsboro, OR, USA (HV 2kV, magnification 40,000×). Imaging was performed using an ETD detector. After the observation of three biological replicates, representative micrographs were taken in triplicate for each magnification. 

### 4.14. EPS Preparation

Free EPSs were precipitated from the culture liquid with ethanol, and hard-to-extract (HE) EPSs were extracted from the membrane with 4 M NaOH, according to the method described by Fang et al., 2015 [10]. The isolated EPSs were freeze-dried and weighed.

### 4.15. Statistical Analysis

For each multiple sample numerical analysis, the results were analyzed for their statistical significance. The analyses were performed by means of T-student test. The results were assumed to be statistically significant when *p* was lower than 0.05.

## 5. Conclusions

In the presented article the effect of *clpP* gene disruption on cell growth and the response to various stress factors in bacteria of the genus *Komagataeibacter xylinus* E_25_ is analyzed. We confirmed the hypothesis that ClpP affects the biosynthesis and structure of the BNC membrane. The protein participates in cell protection against stress factors and its absence may possibly simultaneously disrupt multiple related regulatory networks by affecting the transcriptome or protein stability. As a result of these changes, the cells of E_25_ clpP:amp mutant became longer and had an irregular surface. They also became more fragile to various stress conditions, such as elevated temperature, ethanol content, osmotic shock, and H_2_O_2_ presence. The mutated strain produced cellulose more slowly as related to the parent E_25_ strain. The results also indicate that ClpP absence influences the structure of the cellulose fiber network by increasing fiber thickness and the content of HE-EPS between them and at the same time producing more acetate. The detailed analysis of biosynthesis products, including BNC structure assessment, will give us a better insight into the effects of introduced changes. Further research must still be performed in order to confirm the hypothesis of a connection between the mutation in the *clpP* gene and cellulose structure and properties. In time, perspective research on the possibility of influencing the properties of BNC may open up new possibilities of material structure manipulation and its subsequent applications. At this stage, most of all, the studies provide a foundation for future research on the regulatory pathways of the ClpP-related complex in BNC production. They are also important in understanding the protective mechanism of *Komagataeibacter* cells induced by exposure to stress factors.

## Figures and Tables

**Table 1:** Characteristics of cellulose produced from glucose by *K. xylinus* E_25_ and E_25_ clpP:amp.

Strain	Characteristics of Cellulose	Result After 7 Days
E_25_	Density (g/m^3^)	3.617 ± 0.364
	Yield (g/L)	1.451 ± 0.090
	Free EPS (mg/L)	189.0 ± 14.93
	HE-EPS yield (mg/L) (percentage of BNC)	34.00 ± 7.94 (~2.34%)
E_25_ clpP:amp	Density (g/m^3^)	4.873 ± 0.307
	Yield (g/L)	1.429 ± 0.113
	Free EPS (mg/L)	224.0 ± 6.11
	HE-EPS yield (mg/L) (percentage of BNC)	46.5 ± 12.02 (~3.25%)

## Data Availability

The original contributions presented in this study are included in the article/Supplementary Material. Further inquiries can be directed to the corresponding author.

## Supplementary Materials

The following supporting information can be downloaded at https://www.mdpi.com/article/10.3390/ijms262412047/s1.

## Abbreviations

The following abbreviations are used in this manuscript:

BNC	Bacterial Nanocellulose
HS	Hestrin and Schramm medium
EPS	Exopolysaccharide
HE-EPS	Hard-to-extract exopolysaccharide
