# Supplementary material for: The Effect of clpP Gene Disruption on Cell Morphology, Growth, and the Ability to Synthesize Cellulose of Komagataeibacter xylinus E25
Source: Int J Mol Sci. 2025 Dec 15;26(24):12047. doi: 10.3390/ijms262412047 (PMC12733265; doi:10.3390/ijms262412047)
Supplement: Supplementary file 1 [file ijms-26-12047-s001.zip › ijms-3982557-supplementary.pdf]

International Journal of
Molecular Sciences

MDPI

Article

The effect of *clpP* gene disruption on cells morphology, growth, and the ability to synthesize cellulose of *Komagataeibacter xylinus* E_{25}

Marzena Jedrzejczak-Krzepkowska[1*], Karolina Ludwicka[2*] and Stanislaw Bielecki[3]

Institute of Molecular and Industrial Biotechnology, Lodz University of Technology, Stefanowskiego Street 2/22, 90-537 Lodz, Poland; stanislaw.bielecki@p.lodz.pl
* Correspondence: marzena.jedrzejczak-krzepkowska@p.lodz.pl (M.J.-K.); karolina.ludwicka@p.lodz.pl (K.L.)

Supplementary Materials:

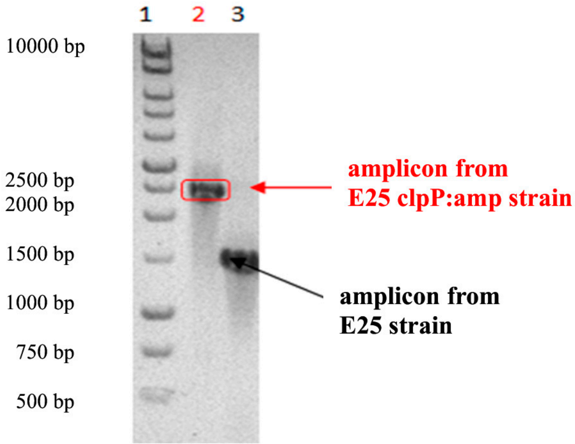

Figure S1. PCR analysis of E_{25} clpP:amp mutant genomic DNA. PCR identification of E_{25} clpP:amp mutant applied flankF and flankR primers. Lane 1 – molecular marker (10,000-base pair DNA ladder); lane 2 – wild-type E_{25} strain; lane 3 – E_{25} clpP:amp strain disrupted with amp resistance cassette.

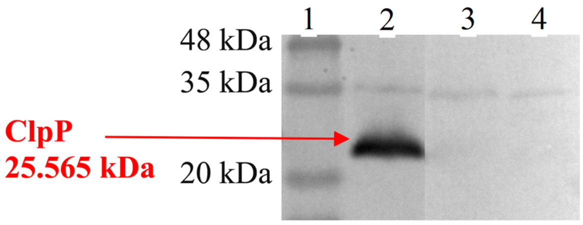

Figure S2. Western blot analysis of cell-free extracts performed using anti-His antibody. Lanes: 1 - molecular weight markers, 2 - E_{25}C; 3 – E_{25} (control), 4 – E_{25} clpP:amp mutant.

 https://doi.org/10.3390/ijms262412047

Table S1. Bacterial strains, plasmids and primers used in this study. In the primer sequence, the restriction site for BssHII enzyme is marked in yellow, red – the restriction site for BspHI , green – restriction site for XhoI, purple – restriction site for HindIII, and bold blue – sequence complementary to 6 histidine tags.

Strains, plasmids, Primers	Characteristics or sequence	Source or reference
**Strains**		
Escherichia coli DH5α	*F⁻ Φ80lacZΔM15 Δ(lacZYA-argF)U169 recA1 endA1 hsdR17(irk⁻, mk⁺) phoA supE44 thi-1 gyrA96 rel A1 λ⁻*	Invitrogen
K. xylinus E_{25}	wild type	derived from ITB TUL, bought by BOWIL Biotech Sp. z o.o.
K. xylinus E_{25} clpP:amp	*K. xylinus* E_{25} mutant with disrupted *clpP* gene	This study
K. xylinus E_{25}C	*K. xylinus* E_{25} clpP:amp, containing pBBR122-clpP	This study
**Plasmids**		
pBBR122	Broad Host Range Vector; Cm^R i Kan^R	MoBiTec, Germany
pBBR122-clpP	pBBR122, containing *clpP* gene for ΔclpP, Cm^R	This study
pEt14b	cloning vector	Novagen
pUC19	cloning vector	Invitrogen, USA
pET14b-clpP-ampR	pET14b, containing *clpP* gene disrupted by a DNA fragment (promoter amp i amp gen)	This study
**Primers**		
pBla-AmpF/BssHII	TTGGCGCGCACCCCTATTTGTTTATTTTTCTAA	This study
pBla-AmpR/BssHII	TTGGCGCGCTTACCAATGCTTAATCAGTGA	This study
clpPF	AGCTCATGATGGCTATGAGGGATCGG	This study
clpPR	CGCTCATGATTACTCCGCAGGTTTTGAC	This study
flankF	GAACAGGCCGTGTTCGAGTAC	This study
flankR	GGCGCTTGTAGTGGTTATGC	This study
clpPHF	CCGCTCGAGTATGGCTATGAGGGATCG	This study
